# Correlation Between Chemical Composition and Antibacterial Activity of Essential Oils from Fifteen Eucalyptus Species Growing in the Korbous and Jbel Abderrahman Arboreta (North East Tunisia)

**DOI:** 10.3390/molecules17033044

**Published:** 2012-03-12

**Authors:** Ameur Elaissi, Zyed Rouis, Samia Mabrouk, Karima Bel Haj Salah, Mahjoub Aouni, Mohamed Larbi Khouja, Farhat Farhat, Rachid Chemli, Fethia Harzallah-Skhiri

**Affiliations:** 1 Laboratory of Pharmacognosy, Faculty of Pharmacy, University of Monastir, Avenue Avicenne, Monastir 5019, Tunisia; Email: rachid_chemli@yahoo.com; 2 Laboratory of Transmissible Diseases and Biologically Active Substances, Faculty of Pharmacy, University of Monastir, rue Avicenne, Monastir 5000, Tunisia; Email: zyedrou@yahoo.fr (Z.R.); karimabhs@yahoo.fr (K.B.H.S.); Ouni@fphm.rnu.tn (M.A.); 3 Laboratory of Genetic, Biodiversity and Bio-resources Valorisation, Higher Institute of Biotechnology of Monastir, University of Monastir, Avenue Tahar Haddad, Monastir 5000, Tunisia; Email: telelsa@yahoo.fr (S.M.); fethiaprosopis@yahoo.fr (F.H.S.); 4 National Institute for Research on Rural Engineering, Water and Forestry, Institution of Agricultural Research and Higher Education, BP. N.2, Ariana 2080, Tunisia; Email: khouja.medlarbi@iresa.agrinet.tn; 5 Laboratory of Analytical Chemistry, Faculty of Pharmacy, University of Monastir, Avenue Avicenne, Monastir 5019, Tunisia; Email: farhat.farhat@fphm.rnu.tn

**Keywords:** antibacterial activity, *Eucalyptus* leaf essential oil composition, principal components analysis (PCA), hierarchical cluster analysis (HCA)

## Abstract

The essential oils of fifteen *Eucalyptus* species harvested from the Jbel Abderrahman and Korbous arboreta (North East Tunisia) were screened for their antibacterial activities by the agar disc diffusion method. Eighteen major components as identified by GC/FID and GC/MS were selected for a study of the chemical and biological activity variability. The main one was 1,8-cineole, followed by spathulenol, *trans*-pinocarveol, *α*-pinene, *p*-cymene, globulol, cryptone, *β*-phellandrene, viridiflorol, borneol, limonene and isospathulenol. The chemical principal component analysis identified five species groups and subgroups, where each group constituted a chemotype, however that of the values of zone diameter of the inhibition (zdi) identified six groups of *Eucalyptus* oils, characterized by their antibacterial inhibition ability. The strongest activity was shown by *E. platypus* oil against *Enterococcus faecalis* and by *E. lamannii* oil against *Staphylococcus aureus*, *Pseudomonas aeruginosa* and *Escherichia coli*. A correlation between the levels of some major components and the antibacterial activities was observed.

## 1. Introduction

The *Eucalyptus*, a native genus from Australia, belongs to the *Myrtaceae* family and comprises about 800 species [1]. More than 300 species of this genus contain volatile oils in their leaves, although fewer than 20 of these have ever been exploited commercially for the production of essential oils rich in 1,8-cineole (more than 70%) which are used in the pharmaceutical and cosmetic industries [2]. Leaf extracts of *Eucalyptus* have also been approved as food additives [3]. In fact, for many years there has been intense interest in essential oils as a source of natural products. They have been screened for their potential uses as alternative remedies for the treatment of many infections and as natural food preservatives [4,5]. A number of studies have demonstrated the antimicrobial properties of *Eucalyptus* species essential oils against a wide range of microorganisms. The most studied were those from *E. globulus* [6,7], *E. camaldulensis*, *E. tereticornis* and *E. citriodora* [6]. Only a few studies have investigated their activity against pathogenic and food spoilage bacteria [8,9]. *Eucalyptus* essential oils rich in 1,8-cineole (≥70%) have been used by inhalation in pulmonary infections [10]. Some species of *Eucalyptus* have been used in Tunisian folk medicine as an antiseptic of the lower and upper respiratory tracts. In 1957, Tunisia introduced 117 species of Eucalyptus which have essentially been used as firewood, for the production of mine wood and in the fight against erosion [11]. In our previous investigation we have studied the oil chemical composition of the leaves of 56 *Eucalyptus* species developed in Tunisia, harvested from the Hajeb Layoun, Souinet, Korbous, Djebel Abderrahman, and Zerniza arboreta [12,13,14,15,16,17]. In this paper the essentials oils of the leaves of 15 *Eucalyptus* species harvested from the Jbel Abderrahamn and Korbous arboreta (North East Tunisia), whose the chemical composition was previously studied by Elaissi *et al*. [15,16], were screened for their antibacterial activity against four bacteria models. Moreover, we have studied the correlation between their major components and their putative antibacterial activity.

## 2. Results and Discussion

### 2.1. Chemical Composition

The chromatographic analysis [GC (RI) and GC/MS] of the essential oils allowed the identification of 138 components, representing 74.0 to 99.1% of the total oil content [15,17]. Eighteen major compounds with an average concentration greater than 5% have been retained for the statistical analysis (Table 1). The main components were oxygenated monoterpenes, among which 1,8-cineole (2.3 ± 2.1–59.3 ± 10.0%) was the major one in eleven leaf essential oils, followed by *trans*-pinocarveol (0.0–26.0 ± 0.0%), *α*-terpineol (0.0–8.7 ± 2.31%), phellandral (0.0–4.3 ± 0.6%), pinocarvone (0.0–4.0 ± 1.7%) and thymol (0.0–3.7 ± 2.5%). 

Table 1Content [%] of 18 compounds selected for the Principal Component and the Hierarchical Cluster Analyses in the essential oils extracted from the leafs of 15 *Eucalyptus* species.

**Table:** 

Compounds andabbreviations	KI ^a^	Content in %						
		*Eucalyptus* species						
		*E. ast* ^c^	*E. cam*	*E. div*	*E. dun*	*E. fal*	*E. glo*	*E. gom*
*α*-Pinene (α-pin)	1053	21.3 ± 4.2	- ^b^	3.3 ± 3.2	23.7 ± 4.5	6.0 ± 1.7	12.0 ± 3.0	0.7 ± 0.6
Limonene (lim)	1209	1.3 ± 0.6	0.3 ± 0.6	1.0 ± 0.0	2.0 ± 1.0	0.7 ± 0.6	2.3 ± 1.5	-
*β*-Phellandrene (β-phe)	1215	-	-	-	1.0 ± 1.7	-	-	-
1,8-Cineole (1,8-cin)	1218	43.7 ± 4.9	3.7 ± 2.1	37.0 ± 12.1	44.7 ± 2.1	30.7 ± 4.0	53.7 ± 3.2	6.3 ± 2.1
*p*-Cymene (p-cym)	1282	1.0 ± 0.0	11.7 ± 4.0	0.7 ± 0.6	1.0 ± 0.0	-	1.0 ± 0.0	3.0 ± 2.0
Pinocarvone (pin)	1590	1.7 ± 0.6	-	1.0 ± 1.0	1.3 ± 0.6	4.0 ± 0.0	1.7 ± 1.2	3.3 ± 1.5
Terpinene-4-ol (ter-4-ol)	1618	-	2.0 ± 0.0	0.7 ± 0.6	-	-	-	3.7 ± 1.5
Aromadendrene (aro)	1625	3.0 ± 1.0	0.3 ± 0.6	3.0 ± 2.0	1.3 ± 0.6	2.0 ± 0.0	3.7 ± 2.1	1.0 ± 0.0
*trans*-Pinocarveol (tr-pin)	1675	7.7 ± 2.3	-	7.0 ± 4.6	3.7 ± 2.1	26.0 ± 0.0	3.7 ± 1.5	12.3 ± 5.1
Cryptone (cry)	1695	-	12.7 ± 1.2	-	-	-	-	-
*α*-Terpinol (α-ter)	1713	1.3 ± 0.6	-	2.7 ± 2.1	1.3 ± 0.6	2.0 ± 0.0	3.3 ± 2.1	7.3 ± 2.3
Borneol (bor)	1720	-	-	0.7 ± 0.6	-	1.0 ± 0.0	-	2.0 ± 0.0
Phellandral (phe)	1747	-	3.7 ± 0.6	-	-	-	-	-
Globulol (glo)	2103	5.7 ± 1.2	1.0 ± 1.0	6.3 ± 5.5	4.3 ± 0.6	7.0 ± 1.7	4.7 ± 1.2	7.7 ± 1.2
Viridiflorol (vir)	2113	1.0 ± 0.0	1.0 ± 0.0	6.7 ± 8.1	1.7 ± 1.2	1.7 ± 1.2	1.0 ± 0.0	1.0 ± 0.0
Spathulenol (spa)	2151	1.0 ± 0.0	28.0 ± 7.9	0.3 ± 0.6	1.7 ± 1.2	1.3 ± 0.6	-	1.0 ± 0.0
Thymol (thy)	2172	-	1.0 ± 0.0	-	-	-	-	-
Isospathulenol (iso)	2259	-	-	-	-	-	2.0 ± 3.5	-

**Table:** 

Compounds andabbreviations	KI ^a^	Content in %							
					*Eucalyptu*s species				
		*E. kit*	*E. leh*	*E. leu*	*E. mac*	*E. pla*	*E. pol*	*E. pop*	*E. rud*
*α*-Pinene	1053	9.7 ± 8.0	17.7 ± 7.2	7.7 ± 2.3	1.0 ± 0.0	9.3 ± 0.6	1.5 ± 0.7	2.0 ± 1.0	0.7 ± 0.6
Limonene (lim)	1209	1.0 ± 1.0	4.3 ± 0.6	2.3 ± 0.6	3.5 ± 0.7	0.3 ± 0.6	1.5 ± 0.7	1.3 ± 0.6	0.7 ± 0.6
*β*-Phellandrene (β-phe)	1215	-	-	-	-	-	-	-	7.3 ± 3.1
1,8-Cineole (1,8-cin)	1218	4.7 ± 3.1	57.0 ± 4.4	59.3 ± 10.0	34.5 ± 2.1	22.7 ± 4.7	58.0 ± 12.07	47.0 ± 9.2	2.3 ± 2.1
*p*-Cymene (p-cym)	1282	6.7 ± 8.1	2.0 ± 0.0	3.0 ± 3.5	1.0 ± 0.0	7.7 ± 4.0	2.5 ± 2.1	0.7 ± 0.6	16.7 ± 1.5
Pinocarvone (pin)	1590	4.0 ± 1.7	-	1.0 ± 0.0	-	2.3 ± 0.6	0.5 ± .0.7	1.0 ± 1.0	-
Terpinene-4-ol (ter-4-ol)	1618	0.3 ± 0.6	-	-	1.0 ± 0.0	1.3 ± 0.6	0.5 ± 0.7	0.7 ± 0.6	2.0 ± 0.0
Aromadendrene (aro)	1625	1.3 ± 0.6	-	2.3 ± 1.5	-	1.0 ± 1.0	-	2.3 ± 1.2	0.7 ± 0.6
*trans*-Pinocarveol (tr-pin)	1675	21.7 ± 10.0	1.0 ± 0.0	4.7 ± 1.2	-	8.3 ± 2.5	1.5 ± 0.7	6.7 ± 5.5	-
Cryptone (cry)	1695	-	-	-	-	-	1.5 ± 2.1	-	7.0 ± 3.5
*α*-Terpinol (α-ter)	1713	4.7 ± 1.2	8.7 ± 2.3	1.7 ± 0.6	1.0 ± 0.0	1.3 ± 0.6	6.5 ± 0.7	2.7 ± 0.6	1.0 ± 0.0
Borneol (bor)	1720	4.7 ± 0.6	0.7 ± 0.6	-	-	-	-	0.7 ± 1.2	-
Phellandral (phe)	1747	0.3 ± 0.6	-	-	-	-	1.0 ± 0.0	-	4.3 ± 0.6
Globulol (glo)	2103	9.0 ± 7.8	0.7 ± 0.6	6.0 ± 2.6	2.0 ± 0.0	6.3 ± 2.1	2.0 ± 0.0	12.7 ± 1.5	0.7 ± 0.6
Viridiflorol (vir)	2113	1.3 ± 0.6	-	0.7 ± 0.6	-	3.7 ± 1.5	1.0 ± 0.0	2.0 ± 0.0	-
Spathulenol (spa)	2151	1.7 ± 0.6	1.0 ± 0.0	-	1.0 ± 0.0	11.0 ± 6.0	4.5 ± 3.5	-	19.7 ± 4.7
Thymol (thy)	2172	-	-	-	1.5 ± 0.7	0.3 ± 0.6	-	-	3.7 ± 2.5
Isospathulenol (iso)	2259	-	-	-	-	1.3 ± 0.6	0.5 ± 0.7	-	1.3 ± 0.6

^a^ KI: Kovats index determined on Supelcowax medium polar column (BP-20); ^b^ -: Not detected; ^c^ *E. ast: E. astringens; E. cam: E. camaldulensis; E. div: E. diversifolia; E. dun: E. dundasii; E. fal: E. falcate; E. glo: E. globules; E. gom: E. gomphocephala; E. kit: E. kitsoniana; E. leh: E. lehmannii; E. leu: E. leucoxylon; E. mac: E. maculate; E. pla: E. platypus; E. pol: E. polyanthemos; E. pop: E. populifolia; E. rud: E. rudis*.

### 2.2. Principal Components Analysis (PCA) and Hierarchical Cluster Analysis (HCA)

**Figure 1:**
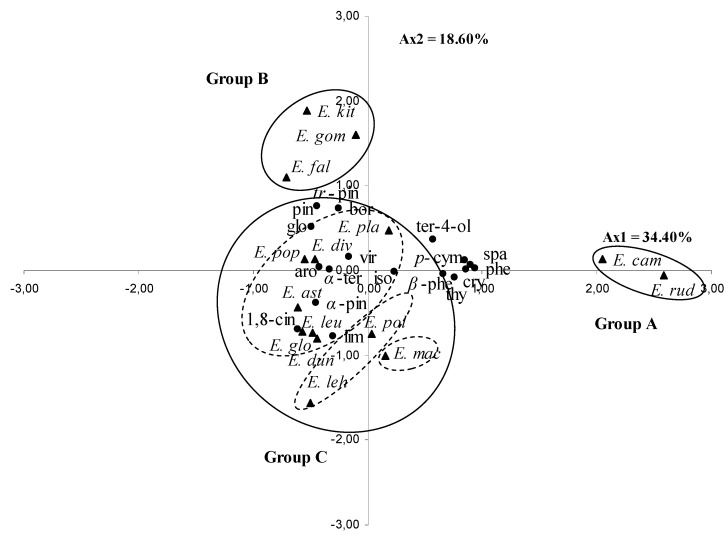
Principal component analysis of 18 major compounds of leaf essential oils of 15 *Eucalyptus* species. For the abbreviation of the *Eucalyptus* species (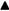) and the compounds ( 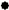), see Table 1.

The contents of the 18 selected leaf essential oil components were differed significantly (*p* < 0.05) between the species (Table 1). These 18 components were used for the PCA and the HCA. The PCA horizontal axis explained 34.40% of the total variance while the vertical axis a further 18.60% (Figure 1). 

**Figure 2:**
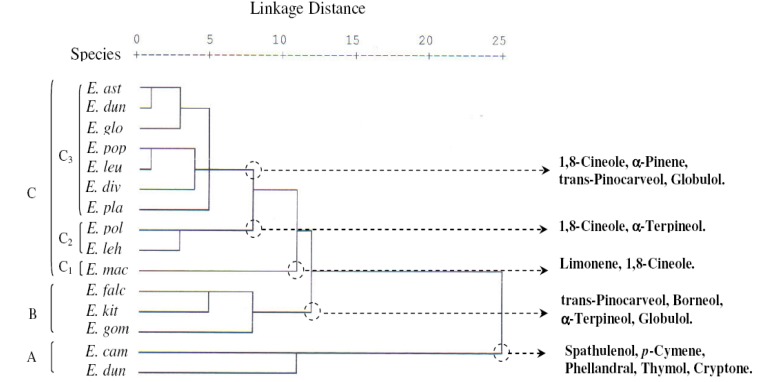
Dendrogram obtained by hierarchical cluster analysis based on the Euclidean distance between groups of the leaf essential oils of fifteen Tunisian *Eucalyptus* species. Components that characterize the major subgroups, considered as chemotypes, are indicated. For the abbreviation of the *Eucalyptus* species, see Table 1.

**Figure 3:**
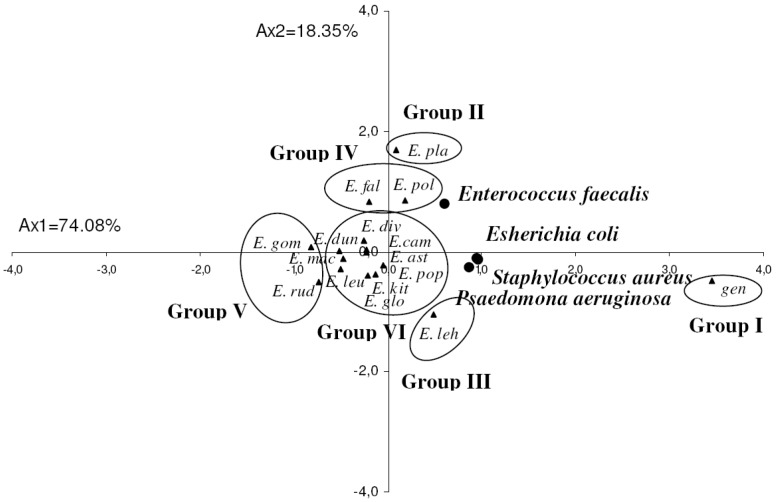
Principal component analysis of the antibacterial activity of 15 *Eucalyptus* species oils against four bacteria model. Gen: gentamicine; for the abbreviation for the *Eucalyptus* species (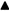) see Table 1.

**Figure 4:**
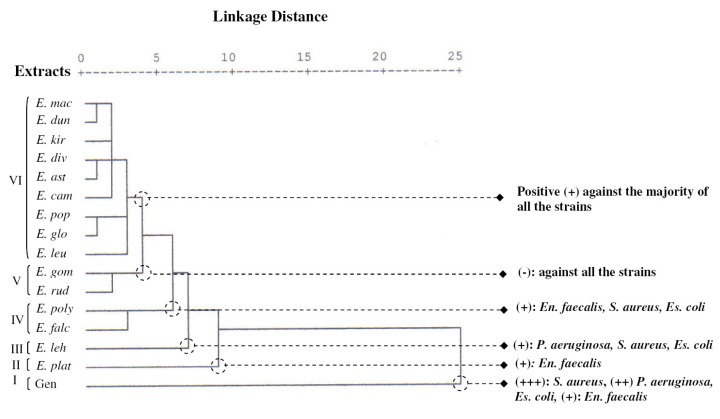
Dendrogram obtained by hierarchical cluster analysis (HCA) based on the Euclidean distance between groups of the leaf essential oils of fifteen Tunisian *Eucalyptus* species and their antibacterial activities against four model strains.

With a dissimilarity of 11.5, the HCA based on the Euclidean distance between groups indicated three groups of species (A, B and C, Figure 2), identified by their essential oil chemotypes. With a dissimilarity > 7, the group C was further divided into three sub-groups (C1, C2 and C3). The Group A and B species clearly stood out forming separate groups in the PCA (Figure 3) and a deep dichotomy in the HCA (Figure 4). The horizontal axis was positively and negatively correlated with group A species and negatively correlated with group B species. The vertical axis was correlated negatively with subgroup C1, subgroup C2 and positively with group B; however the Subgroup C3 occupied the centre of the two axes.

Group A species comprised *E. rudis* and *E. camaldulensis*, with essential oils characterized by the lowest mean percentages of 1,8-cineole (2.3 ± 2.1%, 3.7 ± 2.1% respectively), *trans*-pinocarveol (0.0%), pinocarvone (0.0%), viridiflorol (0.0, 1.0 ± 0.0%) and borneol (0.0%) and by the highest levels of spathulenol (19.7 ± 4.7%, 28.0 ± 7.9%), *p*-cymene (16.7 ± 1.5%, 11.7 ± 4.0%), phellandral (4.3 ± 0.6%, 3.7 ± 0.6%), thymol (3.7 ± 2.5%, 1.0 ± 0.0%), terpinene-4-ol (2.0 ± 0.0%) and cryptone (7.0 ± 3.5%, 12.7 ± 1.2%). We also noted the richness in *β*-phellandrene of *E. rudis* essential oil (7.3 ± 3.1%).

Group B was represented by *E. gomphocephala*, *E. kitsoniana*, *E. falcata*, whose oils were characterized by the highest percentages of *trans*-pinocarveol (12.3 ± 5.1%, 21.7 ± 10.0%, 26.0 ± 0.0%, respectively), pinocarvone (3.3 ± 1.5%, 4.0 ± 1.7%, 4.0 ± 0.0%), borneol (2.0 ± 0.0%, 4.7 ± 0.6%, 1.0 ± 0.0%) and by a relatively high level of *α*-terpineol (7.3 ± 2.3%, 4.7 ± 1.2%, 2.0 ± 0.0%) and globulol (7.7 ± 1.2%, 9.0 ± 7.8%, 7.0 ± 1.7%). The main variation between the three species was due to the richness of *E. falcata* oil in 1,8-cineole (30.7 ± 4.0%) against 4.7 to 6.3% for the two others species, and to the poverty of *E. gomphocephala* oil in α-pinene (0.7 ± 0.6%) which was found with a higher content in those of *E. falcata* and *E. kitsoniana* oils (6.0 ± 1.7%, 9.7 ± 8.0%, respectively).

Sub-group C1 was reduced to *E. maculate*, whose essential oil was characterized by only the two components: limonene and 1,8-cineole (3.5 ± 0.7%, 34.5 ± 2.1%, respectively), while the mean percentage of all the other compounds was very low (0–1.5%).

Subgroup C2, represented by *E. lehmannii* and *E. polyanthemos*, was characterized by oils containing a high mean percentage of 1,8-cineole (57.0 ± 4.4%, 58.0 ± 12.07%, respectively) and *α*-terpineol (8.7 ± 2.3%, 6.5 ± 0.7%, respectively). The *E. lehmannii* oil was richer in *α*-pinene and limonene, whereas the *E. polyanthemos* oil was richer in spathulenol.

Subgroup C3 contained seven species: *E. platypus*, *E. diversifolia*, *E. leucoxylon*, *E. populifolia*, *E. globulus*, *E. dundasii* and *E. astringens*, which were characterized by oils with a high mean percentage of 1,8-cineole (22.7 ± 4.7–59.3 ± 10.0%), a moderate content of *α*-pinene (3.3 ± 3.2–23.7 ± 4.5%), *trans*-pinocarveol (3.7 ± 1.5–8.3 ± 2.5%), globulol (4.3 ± 0.6–12.7 ± 1.5%) and a low content of limonene, *β*-phellandrene, *p*-cymene, pinocarvone, borneol and thymol, with an exception for *E. platypus* which contained a relatively moderate percentage of *p*-cymene (7.7 ± 4.0%) and isospathulenol (1.3 ± 0.6%).

### 2.3. Antibacterial Activity

The essential oils were tested at 10 μL per disc for their putative antibacterial activity against four bacterial species; the results are shown in Table 2. According to the zone diameter inhibition (zdi) values expressed in mm, results were ranked as follows: not sensitive (–) for zone diameters equal to 8 mm or below; sensitive (+) for zone diameters between 8 and 14 mm, very sensitive (++) for zone diameters between 14 and 20 mm and extremely sensitive (+++) for zone diameters equal or larger than 20 mm [18,19,20]. To evaluate the correlations between the antibacterial activities and the essential oils of the 15 *Eucalyptus* species studied, all the mean values of the zone diameters inhibition were subject to PCA and HCA analyses. The statistical analysis of the antibacterial activities of the oils showed a significant difference (*p* < 0.05) between *Eucalyptus* species oils and the different bacteria strains. 

The PCA horizontal axis explained 74.08% of the total variance, while the vertical axis a further 18.35% (Figure 3). The HCA indicated six groups of species (I, II, III, IV, V and VI) identified by their bacteria growth inhibition with a dissimilarity ≥3.0 (Figure 4). The horizontal axis correlated positively with group I and negatively with group V and presented the maximum of the variation, however vertical axis was correlated negatively with group II and positively with group III and IV. The first principal axis separated group I from group V, however the second principal axis separated group II from groups III and IV, while group VI occupied the centre of the two principal axes. The variation between its species was therefore very low.

The first group (Group I) was constituted by the standard antibiotic gentamicine. It stood out forming a separate group in HCA and PCA. All the strains were very sensitive to this antibiotic, which showed the higher zone diameter of inhibition (13.0 ± 1.0–29.3 ± 1.2 mm).

Table 2Antimicrobial activity of the investigated essential oils and the standard antibiotic (gentamicin) against four model bacteria.

**Table:** 

Microorganisms	Inhibition zone diameters ^♣^					
	Essential oils					
	*E. ast*	*E. cam*	*E. div*	*E. dun*	*E. fal*	*E. glo*
Gram^+^						
*Enterococcus faecalis*	9.0 ± 1.0 ^a^^°^	9.0 ± 1.0 ^a^	9.3 ± 0.6 ^ab^	8.7 ± 2.1 ^a^	10.3 ± 2.1 ^abc^	8.7 ± 0.6 ^a^
*Staphylococcus aureus*	9.3 ± 1.5 ^abcd^	11.7 ± 0.6 ^de^	8.3 ± 2.5 ^ab^	8.0 ± 0.0 ^ab^	11.3 ± 3.8 ^cde^	9.0 ± 0.0 ^abcd^
Gram^−^						
*Escherichia coli*	8.3 ± 0.6 ^bcd^	7.3 ± 0.6 ^abc ^	8.3 ± 0.6 ^bcd ^	7.0 ± 1.0 ^ab^	8.0 ± 2.0 ^bc^	8.0 ± 0.0 ^bc^
*Pseudomonas aeruginosa*	7.3 ± 0.6 ^ab^	7.0 ± 0.0 ^ab^	7.3 ± 0.6 ^ab^	7.0 ± 0.0 ^ab^	6.0 ± 0.0 ^a^	8.7 ± 0.6 ^b^

**Table 2.** *Cont*.

Microorganisms	Inhibition zone diameters				
	Essential oils				
	*E. gom*	*E. kit*	*E. leh*	*E. leu*	*E. mac*
Gram^+^					
*Enterococcus* *faecalis*	8.3 ± 0.6 ^a^	8.3 ± 0.6 ^a^	8.3 ± 0.6 ^a^	8.3 ± 2.3 ^a^	8.5 ± 0.7 ^a^
*Staphylococcus* *aureus*	7.3 ± 0.6 ^a^	10.3 ± 1.5 ^bcd^	12.7 ± 0.0 ^e^	7.0 ± 0.0 ^a^	9.0 ± 0.0 ^abcd^
Gram^−^					
*Escherichia coli*	6.0 ± 0.0 ^a^	8.0 ± 1.7 ^bc^	10.0 ± 0.0 ^d^	6.7 ± 0.6 ^ab^	7.0 ± 0.0 ^ab^
*Pseudomonas aeruginosa*	6.0 ± 0.0 ^a^	7.7 ± 1.2 ^ab^	10.3 ± 0.6 ^c^	8.0 ± 1.0 ^b^	7.0 ± 0.0 ^ab^

**Table:** 

Microorganisms	Inhibition zone diameters				
	Essential oils				
	*E. pla*	*E. pol*	*E. pop*	*E. rud*	Gen ^°^
Gram^+^					
*Enterococcus faecalis*	12.7 ± 5.5 ^cd^	11.1 ± 1.4 ^abc^	9.0 ± 1.0 ^a^	7.3 ± 1.2 ^a^	13.0 ± 1.0 ^d^
*Staphylococcus* *aureus*	8.0 ± 0.0 ^ab^	11.5 ± 2.1 ^cde^	9.3 ± 1.2 ^abcd^	8.7 ± 1.2 ^abc^	29.3 ± 1.2^f^
Gram^−^					
*Escherichia coli*	7.3 ± 0.0 ^ab^	9.0 ± 0.0 ^cd^	8.3 ± 1.5 ^bcd^	7.0 ± 0.0 ^ab^	20.0 ± 1.0 ^e^
*Pseudomonas aeruginosa*	8.7 ± 0.6 ^b^	7.5 ± 0.7 ^ab^	8.7 ± 2.1 ^b^	6.0 ± 0.0 ^a^	14.3 ± 1.5 ^e^

^♣^ The diameter of the disc (φ = 6 mm) was included; ^°^ values with different letters differ significantly by Duncan’s multiple range test (*p* < 0.05); ^°^ Gentamicine 10 μg/disc.

On the other hand the essential oils of *E. rudis* and *E. gomphocephala* of the group V, which was opposite to the group I, produced the lowest zone diameter inhibition and were the least active against the four strains tested. These oils were considered as inactive against the studied bacteria (6.0 ± 0.0–8.7 ± 1.2 mm zdi).

Group II was reduced to *E. platypus*, whose essential oil exhibited the best activity against the Gram positive *Enterococcus faecalis* (12.7 ± 5.5 mm). This activity was comparable to that produced by gentamicine. It was also characterized by a weak inhibition against the other three strains.

Group III included *E. lehmannii* oil, and possessed moderate activity against the Gram negative bacteria *P. aeruginosa* and *Escherichia coli* and the Gram positive *S. aureus*, with inhibition zone diameters equal to 10.3 ± 0.6, 10.0 ± 0.0, and 12.7 ± 0.6 mm, respectively. *E. lehmannii* oil activity was considered as the strongest one among those exhibited by the other studied oils; however it remaind lower than that produced by the standard antibiotic gentamicine. It was also observed that *Enterococcus faecalis* was less sensitive to this oil than the other bacteria (8.3 ± 0.6 mm zdi).

*E. falcata* and *E. polyanthemos* oils of the group IV, were moderately active against the Gram positive bacteria *Enterococcus faecalis* (10.3 ± 2.1, 11.0 ± 1.4 mm zdi, respectively) and *S. aureus* (11.3 ± 3.8, 11.5 ± 2.1 mm zdi, respectively) and the Gram negative bacteria *Escherichia coli* (8.0 ± 2.0, 9.0 ± 0.0 mm zdi, respectively); whereas the Gram negative *P. aeruginosa* was the most resistant to these oils.

Group VI was made up of *E. leucoxylon*, *E. globulus*, *E. populifolia*, *E. camaldulensis*, *E. astringens*, *E. diversifolia*, *E. kitsoniana*, *E. dundasii* and *E. maculata* oils, with no significant difference in their activity against all the strains (6.7 ± 0.6 ≤ zdi ≤ 9.3 ± 1.5 mm); however *E. kitsoniana* and *E. camaldulensis* oils showed a moderate inhibition against the Gram positive *S. aureus* (10.3 ± 1.5, 11.7 ± 0.6 mm, zdi, respectively).

The antimicrobial activity of the essential oils displayed a considerable variation among the different *Eucalyptus* species oils. In general the zone diameter inhibition was lower than that exhibited by the standard antibiotic gentamicine, but we noticed some very favourable comparable results, especially with the oil of *E. platypus* against *Enterococcus faecalis*. This variability could be attributed to their different chemical composition. The lowest activity was mostly evident with the essential oils of *E. rudis* and *E. gomphocephala* belonging to different chemotypes. These results could be explained by their lack of 1,8-cineole (2.3 ± 2.1, 6.3 ± 2.1%, respectively) which helps to permeabilize membranes, allowing the more active terpinene to enter and kill the bacterial cell [21]. In fact their essential oils were rich in other compounds such as *p*-cymene, spathulenol, cryptone for *E. rudis* and *trans*-pinocarveol, *α*-terpineol, globulol for *E. gomphocephala* which could be by antagonism be responsible for the lower activity. However the comparison between the activities of oils from the same chemotype, such as those of *E. gomphocephala*, *E. kitsoniana* and *E. falcata* showed that they varied significantly between the species. This variability could be due to the difference in the levels of their major and minor components and to a synergetic effect between all components. This finding was in agreement with Dellacassa *et al*. [22], Zakarya *et al*. [23] and Chalchat *et al*. [24]. The Gram positive bacteria were found more sensitive to these oils than the Gram negative bacteria. Our results confirmed the observations of Essawi and Sorour [25] proving that *S. aureus*, having a single layer wall, was the most sensitive to essential oils as compared to *Escherichia coli* which has a multilayer membrane structure. The highest activity corresponded to *E. lehmannii* oil against *Escherichia coli*, *S. aureus*, *P. aeruginosa* and *E. platypus* against *Enterococcus faecalis*. The two species share a high mean percentage of 1,8-cineole, whereas the of *E. platypus* oil was distinguished by a higher mean percentage of the monoterpene *p*-cymene (7.7 ± 4.0%) and *trans*-pinocarveol (8.3 ± 2.5%). The *E. falcata* oil, which was rich in *trans*-pinocarveol (26.0 ± 0.0%) and in 1,8 cineole (30.7 ± 4.0%) and very poor in *p*-cymene (0.0%) had a lower activity against *Enterococcus faecalis* than that of the *E. platypus* oil. We suggest that the monoterpene hydrocabon *p*-cymene could be one of the components that made the Gram-positive *Enterococcus faecalis* more sensitive. Bakkali, Averbeck, Averbeck, and Idaomar [26] demonstrated that compounds such as linalool, *γ*-terpinene, *p*-cymene, and *α*-terpineol had relatively strong antimicrobial activities. Since the monterpenes *α*-pinene, limonene and *trans*-pinocarveol were more abundant in *E. lehmannii* oil, the activity of the latter against *P. aeruginosa*, *Escherichia coli* and *S. aureus* was correspondingly more important than that of *E. platypus* oil. This allowed us to deduce that these strains were more sensitive to these components. *E. falcata* and *E. polyanthemos* were grouped together in the PCA antibacterial activity, their essential oils being composed essentially by 1,8-cineole, and by other different levels of major and minor compounds, which could be the result a of synergetic effect effective against *E. faecalis* and *S. aureus*. On the other hand, the *E. lehmannii* oil which belonged to the same chemotype as *E. polyanthemos* possessed a stronger activity against the Gram negative bacteria *E. coli* and *P. aeruginosa*. There is evidence that essential oils were mostly strongly antimicrobial than can be accounted for by the additive effect of their major antimicrobial components; we also suggest that minor components appear, therefore, to play a significant role [27]. The majority of group VI species which were characterized by a high level of 1,8-cineole and a moderate content of *α*-pinene and *trans*-pinocarveol reduced the growth level of the four pathogenic bacteria without any significant differences between them. While the *E. camaldulensis* oil belonging to the same antibacterial group, was very poor in 1,8-cineole and relatively rich in the ketone cryptone and in the monoterpene hydrocarbon *p*-cymene, but was more effective against the Gram-positive (*S. aureus*) than the oils of the same antibacterial group. The comparative study of our results to those obtained by Cimanga *et al*. (2002) [6] showed that essential oil of *E. camaldulensis* from Congo which was essentially composed by 1,8-cineole (58.9%),*α*-pinene (5.4%), limonene (5.4%), myrtenal (3.5%), *γ*-terpinene (2.8%), *p*-cymene (2.1%) and cryptone (1.1%), had a better activity against *Escherichia coli* (10.0–12.0 mm, zdi), *P. aeruginosa* (15–16 mm, di) and *S. aureus* (18–30 mm, zdi) than that from Tunisia, which was very poor in 1,8-cineole (3.7%) but relatively richer in *p*-cymene (11.7%) and cryptone (12.7%). The *E. globulus* oil from Tunisia and from the Congo (Cimanga *et al*. [6]) had a similar activity against the Gram negative bacteria *P. aeruginosa*. The oil of Congolese provenance was characterized by 1,8-cineole (44.3%) and *α*-pinene (9.3%) as major components and also by a high level of camphene (23.1%) [6], which might be responsible for the higher sensitivity of the Gram negative *Escherichia coli* to this oil (15–16 mm zdi) then that from Tunisia.

## 3. Experimental

### 3.1. Plant Materials

Samples of clean mature leaves of 15 species of the genus *Eucalyptus* L’HÉR. were picked in April 2007, from 10 species acclimated in the Korbous arboreta (*E. astringens* Maiden, *E. camaldulensis* Dehnh., *E. diversifolia* Bonpl., *E. falcata* Turcz., *E. gomphocephala* DC., *E. lehmannii* (Schauer) Benth., *E. maculata* Hook., *E. platypus* Hook., *E. polyanthemos* Schauer, and *E. rudis* Endl.) and from five species acclimated in the Jbel abderrahman arboreta (*E. dundasii* Maiden., *E. globulus* Labill., *E. kitsoniana* F. Muell., *E. leucoxylon* F.Muell. and *E. populifolia* Hook.). Both of the arboreta were located in the Nabeul region (North East Tunisia with sub-humid bioclimate). Botanical voucher specimens have been deposited in the Pharmacognosy Laborotary Herbarium in the Faculty of Pharmacy, Monastir, Tunisia, under the following references: 0132, 0133, 0134, 0135, 0137, 0138, 0139, 0140, 0141, 0142, 0151, 0152, 0153, 0154 and 0.155.

### 3.2. Extraction of Essential Oils

Extraction was carried out by hydrodistillation during 4 h, using a standard apparatus recommended in the European Pharmacopoeia. We made three attempts for each sample of 100 g of crudely crushed dried leaves and for each species. The oil collected from each plant was dehydrated with Na_2_SO_4_ and stored at 4 °C, until chemical analysis and biological activities were attempted.

### 3.3. Chemical Analysis

Quantitative and qualitative data of all the essential oils were determined in triplicate by GC and GC/MS respectively. 

#### 3.3.1. Gas Chromatography Analysis

GC analysis was carried out with a Hewlett-Packard HP 6890 apparatus equipped with FID and an intermediate polarity SPB-20 capillary column (30 m × 0.32 mm i.d., film thickness 0.25 μm). The oven temperature was programmed isothermally at 35 °C for 1 min, rising from 35 to 250 °C at 5 °C/min, and then held isothermal at 250 °C for 3 min; injector temp., 250 °C; detector temp., 280 °C; carrier gas, N_2_ (1.2 mL/min). The injected volume was 1 μL (10% essential oil in purified hexane). The relative concentration was calculated using the HP Chemstation software, which allowed the assimilation of the percentages of the peak areas to the percentages of the various constituents. Retention indices (RI) were determined relatively to the retention times (tR) of a series of *n*-alkanes (C9–C28).

#### 3.3.2. Gas-Chromatography-Mass-Spectrometry Analysis

All the essential oils were analyzed by GC/MS using a Hewlett-Packard 5890 series II apparatus equipped with a polar Carbowax capillary column (30m × 0.32 mm i.d., film thickness 0.25 μm) and 5972 mass selective detectors. Helium was used as the carrier gas. The mass spectrometer operating conditions were: ionisation voltage, 70 eV; ion source 230 °C. The GC analytical conditions were as described above (see GC analysis).

#### 3.3.3. Compound Identification

The identification of the compounds was based on a comparison of their retention indexes determined relative to the retention time of aliphatic hydrocarbons (C9–C28) and of the mass spectra with those of authentic compounds by means of NBS75K.L. and Wiley 275 databases and with the literature data [28]. 

### 3.4. Antibacterial Testing

The antibacterial activity of the different essential oils was evaluated by the paper-disc agar diffusion method against the two Gram-negative model bacteria *Escherichia coli* (ATCC 25922) and *Pseudomonas aeruginosa* (ATCC 27853) and the two Gram-positive bacteria *Staphylococcus aure* us (ATCC 25923) and *Enterococcus faecalis* (ATCC 29212). Microorganisms were obtained from the culture collection of the Laboratory of Transmissible Disease and Biologically Active Substances, Faculty of Pharmacy of Monastir, Tunisia. Organisms were maintained on Muller-Hinton agar (MH) (BIORAD) medium. Inocula were prepared by diluting overnight (24 h at 37 °C) cultures in Muller Hinton Broth medium to approximately 106 CFU/mL. Absorbent discs (Whatman N°3 discs, 6 mm diameter) were impregnated with 10 μL of oil and then placed on the surface of inoculated plates (90 mm). Positive control discs of gentamicine (10 μg/disc) were included in each assay. Diameters of growth inhibition zones were measured after incubation at 37 °C for 24 h. The experiment was done in triplicate. 

### 3.5. Statistical Analysis

The data were analyzed using analysis of variance (ANOVA) and the significance of the differences between means was determined at *p* < 0.05 using Duncan's multiple range tests. Results were expressed as means ± standard deviation. To evaluate whether the identified essential oils components may be useful in reflecting chemotaxonomic and biological activities relationships, 18 compounds detected in the oil samples at an average concentration greater than 2% of the total oil were selected and used for this purpose. Both of these components and all the values of the essential oils zones diameters of bacteria growth inhibition were subjected to a principal components analysis (PCA) and to a hierarchical clusters analysis (HCA) using SPSS 12.0 software (SPSS Inc. Chicago, IL, USA).

## 4. Conclusions

The chemical PCA and HCA analysis separated all the species oils into five groups and sub-groups, each group constituted a chemotype; however in that of the antibacterial activity, six groups of *Eucalyptus* oils were identified and separated by their levels of the growth inhibition of the studied bacteria. *E. leucoxylon* of the sub-group C3 was the richest species in 1,8-cineole. The activity of the Eucalyptus *essential* oils varied significantly within species and within strains. In general, the strong antimicrobial activity was related not only to a high content of one major component such as 1,8-cineole, but the presence of the moderate and the minor compounds was indispensable. The Gram positive bacteria *S. aureus* and *Enterococcus faecalis* were the most sensitive, while the Gram negative bacteria *P. aeruginosa* and *Escherichia coli* were the most resistant. *E. platypus oil* showed the best zone diameter inhibition against *E. faecalis*, while *S. aureus*, followed by *P. aeruginosa* and *Escherichia coli*, were more sensitive to *E. lehmannii* oil. These species oils may have an interesting prospect in therapeutic application for treatment of some bacterial infections.
